# Recruiting the innate immune system with GM-CSF to fight viral diseases, including West Nile Virus encephalitis and COVID-19

**DOI:** 10.12688/f1000research.23729.1

**Published:** 2020-05-11

**Authors:** Huntington Potter, Timothy D. Boyd, Penny Clarke, Victoria S. Pelak, Kenneth L. Tyler

**Affiliations:** 1 Department of Neurology, University of Colorado Anschutz Medical Campus, Aurora, CO, 80045, USA; 2 University of Colorado Alzheimer's and Cognition Center, Aurora, CO, 80045, USA; 3 Linda Crnic Institute for Down Syndrome, Aurora, CO, 80045, USA

**Keywords:** COVID-19, SARS-CoV-2, West Nile Virus, granulocyte-macrophage colony-stimulating factor, Alzheimer’s Disease, inflammation, acute respiratory distress syndrome, neuroinflammation

## Abstract

As the coronavirus disease 2019 (COVID-19) pandemic grows throughout the world, it is imperative that all approaches to ameliorating its effects be investigated, including repurposing drugs that show promise in other diseases. We have been investigating an approach to multiple disorders that involves recruiting the innate immune system to aid the body’s healing and regenerative mechanism(s). In the case of West Nile Virus encephalitis and potentially COVID-19, the proposed intervention to stimulate the innate immune system may give the adaptive immune response the necessary time to develop, finish clearing the virus, and provide future immunity. Furthermore, we have found that GM-CSF-induced recruitment of the innate immune system is also able to reverse brain pathology, neuroinflammation and cognitive deficits in mouse models of Alzheimer’s disease and Down syndrome, as well as improving cognition in normal aging and in human patients with cognitive deficits due to chemotherapy, both of which exhibit neuroinflammation. Others have shown that GM-CSF is an effective treatment for both bacterial and viral pneumonias, and their associated inflammation, in animals and that it has successfully treated pneumonia-associated Acute Respiratory Distress Syndrome in humans. These and other data strongly suggest that GM-CSF may be an effective treatment for many viral infections, including COVID-19.

There is much debate about how to best treat disorders that include an inflammatory component. For example, in Alzheimer’s disease (AD), inflammation is evidenced by the activation of microglia and astrocytes, the consequent over-expression of inflammatory cytokines and acute phase proteins in the brain, and by the presence of inflammatory markers in the blood ^1, 2^. Furthermore, in AD, some inflammatory proteins expressed in the brain, specifically α1-antichymotrypsin and apolipoprotein E (ApoE), especially ApoE4, clearly promote and, indeed, are necessary for amyloidogenesis that leads to cognitive decline in humans and/or animal models ^3, 4^. The finding that the introduction of pro-inflammatory molecules, such as lipopolysaccharide, into mouse models of AD exacerbated amyloid pathology and cognitive deficits also suggested that inflammation contributed essentially to the disease, possibly by a self-perpetuating feed-forward mechanism ^5^. This idea was reinforced by the finding that people with rheumatoid arthiritis, who routinely take non-steroidal anti-inflammatory drugs (NSAIDS) to control the swelling and inflammation in their joints, have a much lower risk of developing AD, which suggested that their NSAID use was protective against, and might be used to treat, AD ^6, 7^.

In the case of viral diseases, including West Nile Virus (WNV) encephalitis, bacterial and viral pneumonia, Severe Acute Respiratory Syndrome-coronavirus (SARS-CoV-1), Middle East Respiratory Syndrome-coronavirus (MERS-CoV), the development of inflammation has been cited as evidence that an effective treatment should include anti-inflammatory drugs, including steroids ^8, 9^. Indeed, steroids have been used in some human patients with WNV infection ^10– 13^. In respiratory diseases, an overactive innate immune reaction, termed a ‘cytokine storm’ may arise and has been treated with high dose steroids. Furthermore, use of minocycline to suppress microglial activation and promote expression of anti-inflammatory cytokines reduces neuronal cytotoxicity and mortality in WNV-infected mice ^14, 15^. Minocycline, has also been suggested as a treatment for AD based on experiments in cell culture and animal models, but its effects were complex and resulting changes to the peripheral and CNS immune system remain unclear ^16, 17^.

Despite the logical and preclinical data-based arguments in favor of using anti-inflammatory drugs for treatment of inflammation-associated disorders, clinical trials showed that NSAIDs were unsuccessful and potentially detrimental in AD and mild cognitive impairment participants ^7, 18– 20^, and minocycline also failed in a recently-completed trial in participants with early AD ^21^. In WNV and Japanese Encephalitis Virus infected mice, *reducing* microglia in the brain by blocking the receptor of Macrophage Colony Stimulating Factor (MCSF) with PLX5622 ^9^ resulted in increased viral load and mortality, suggesting that microglia, traditionally considered to be an indication of a dangerous pro-inflammatory milieu may, in fact be beneficial in fighting the virus infection.

Such alternative, even opposing, views of the role of inflammation is particularly striking in recent discussions about treating COVID-19. For example, it is proposed that inflammation in COVID-19-associated SARS should be suppressed because the cytokine storm is thought to play a major role in the pulmonary damage that so greatly increases morbidity and mortality in SARS (also termed acute respiratory distress syndrome, ARDS), a feature of infection by both SARS-CoV-1 and SARS-CoV-2 ^22^. Recent studies report increased plasma levels of chemokines and cytokines, including CCL-2, CCL-3, RANTES, INFγ, IL-1β, IL-2, IL-6, IL-8, IL-10, G-CSF, IP10, TNFα, and others, in severe cases of COVID-19 as compared to mild cases ^23, 24^, while decreased levels of lymphocytes are also commonly found in severe cases of SARS and COVID-19 patients ^25– 30^. Thus, it is unclear whether treatment with broad-acting immunosuppressant drugs to combat the increased cytokine and chemokine signaling would also exacerbate leukopenia, resulting in inhibited clearance of the virus and repair of tissue damage. Because no controlled trials using NSAIDs or steroids in COVID-19 patients have yet been reported, the CDC has taken a cautious stand, stating that “ *Currently, there are no data suggesting an association between COVID-19 clinical outcomes and NSAID use*” and that “ *Corticosteroids have been widely used in hospitalized patients with severe illness in China ^31– 34^; however, the benefit of corticosteroid use cannot be determined based upon uncontrolled observational data. By contrast, patients with MERS-CoV or influenza who were given corticosteroids were more likely to have prolonged viral replication, receive mechanical ventilation, and have higher mortality ^35– 39^. Therefore, corticosteroids should be avoided unless indicated for other reasons, such as management of chronic obstructive pulmonary disease exacerbation or septic shock.”* ^40^

As another, more targeted approach, it is suggested that inflammation may be best prevented or reduced by using specific monoclonal antibodies to block either the IL-6 receptor ^41– 43^ or the cytokine granulocyte-macrophage colony-stimulating factor (GM-CSF) ^41, 44^. The hypothesis that blocking IL-6 or GM-CSF will be beneficial is based in part on the finding that IL-6 and GM-CSF and/or the cells that express them are increased in the blood of COVID-19 patients ^22, 41^ and on the beneficial results froma preliminary single-arm trial of an IL-6 receptor blocker, which reportedly lowered fever and improved oxygenation in COVID-19 patients ^43^. Lowering IL-6 or other inflammatory cytokines in COVID-19 patients is reasonable, but the timing may be critical as early treatment may be detrimental. A multi-arm study including both early and later stage patients is essential to determine the clinical relevance of such intervention ^30, 42^, especially for anti-IL-6 therapies, such as tocilizumab and sarilumab, as IL-6-mediated inflammation has been shown to play a critical role in effective cellular and humoral immunity against viral infections ^45– 48^. Thus, if anti-IL-6 therapies are used too early to treat patients with COVID-19, this strategy could potentially inhibit viral clearance and/or leave the patients susceptible to recurrent infection with SARS-CoV-2.

As an alternative to treatment approaches designed to block pro-inflammatory cytokine responses, we suggest that COVID-19-associated SARS may be better prevented or treated by instead increasing the activation and numbers of macrophages and microglia, in particular by *increasing* the levels of GM-CSF. This innate immune system stimulant has been approved by the FDA as recombinant human GM-CSF (rHuGM-CSF/sargramostim/Leukine ^®^) for almost 30 years as a therapy to increase white blood cell numbers by stimulating the division and differentiation of hematopoietic stem cells ^49^. Approved indications for such treatment include reducing the incidence of severe and life-threatening infections after chemotherapy, accelerating myeloid reconstitution following hematopoietic cell transplantation (HCT), and treating cases of acute radiation syndrome. The finding of low white blood cell counts in patients with COVID-19 ^23^ suggests that the ability of GM-CSF to increase select leukocyte populations, particularly phagocytes, may be of therapeutic value in COVID-19, especially given GM-CSF’s ability to treat life-threatening infections, and as data from Wuhan, China have shown that half of all COVID-19 patients who died had evidence of secondary infections ^50^. This concept is further supported by a previous study that found that the endogenous levels of GM-CSF were significantly higher in bronchoalveolar lavage fluid from patients with ARDS due to trauma or sepsis who *survived* compared to those who died ^51^, in contrast to the potential deleterious effects of GM-CSF that were inferred from the study cited above ^41^.

These results suggest to us that GM-CSF may be beneficial in the treatment of WNV infection and the inflammation that results, and by implication, other viral infections, potentially including COVID-19. In preliminary experiments, we have found that GM-CSF treatment improves survival in mice infected by footpad injection with the TX02 strain of WNV, from approximately 35% in untreated animals to 80% survival in mice treated with GM-CSF starting the day after viral inoculation (p=0.0034) (Clarke, Potter, Boyd, Tyler, manuscript in preparation). These findings are particularly important, as there are currently no effective treatments for WNV encephalitis, and the long safety history of rHuGM CSF/sargramostim in treating leukopenia and preventing life-threatening infections suggests that these preliminary findings, if confirmed, could quickly be translated into human trials.

Furthermore, the potential benefit of GM-CSF as a pulmonary therapy has been shown directly in several other animal models in which GM-CSF treatment was able to both protect against and treat bacterial and viral pneumonias ^52– 61^, which, like COVID-19, induce a cytokine storm that can lead to respiratory distress and multi-organ failure. Notably, some of these studies showed that inhaled GM-CSF was particularly efficacious ^57, 58^. Inhaled GM-CSF has also been used successfully in humans off-label as a treatment for pulmonary alveolar proteinosis ^62– 65^, and successfully as compassionate treatment for pneumonia-associated ARDS ^66^.

The recent report that injection of mesenchymal stem cells (MSCs) reverses clinical and peripheral manifestations of COVID-19 ^67^ and the fact that GM-CSF is well known to stimulate the mobilization of MSCs from the bone marrow ^68– 70^ provide further support for the argument that GM-CSF administration may effectively treat COVID-19.

It is also relevant that the unusually rapid onset of COVID-19-associated SARS after infection with the SARS-CoV-2 virus may be due to infection and inflammation in the central nervous system (CNS), including in the brain stem, which could contribute to respiratory failure, and neurological symptoms have been reported in COVID-19 patients, such as hypogeusia, hyposmia, neuralgia, dizziness, headache, ataxia, epilepsy, and others ^71– 77^. Additionally, in studies of SARS-CoV-1 and MERS-CoV, the presence of viral neuroinvasion has been identified in both animal studies ^78, 79^ and in post-mortem human brain samples ^80, 81^. Significantly, GM-CSF can easily cross the blood-brain barrier ^82^, and our work with GM-CSF in both animals and humans indicates that it can ameliorate CNS disorders of various kinds that include an inflammatory component. For example, GM-CSF/sargramostim treatment is associated with improved cognition in:1) mouse models of Alzheimer’s disease ^83^, which has since been replicated by others ^84^, 2) a mouse model of Down syndrome (Ahmed, Boyd, Potter *et al*., manuscript in preparation), 3) aged wild-type control mice ^83^ and (Ahmed, Boyd, Potter *et al*., manuscript in preparation), which has also been replicated by others ^85^, 4) a retrospective study of leukemia patients after immune system chemoablation and HCT, who acquire chemotherapy-associated cognitive impairment ^86^, and 5) mild-to-moderate Alzheimer’s disease participants, based on one measure, the Mini-Mental State Exam (recombinant human GM-CSF/sargramostim, five days/week for three weeks (Potter, Woodcock, Boyd *et al*., NCT01409915; manuscript submitted), all of which are CNS disorders. These findings are relevant to COVID-19 because all of these disorders induce inflammation ^1, 8, 9^ and yet still benefit from treatment with GM-CSF, which *increases*, rather than decreases, the number and activation of microglia, the resident phagocytes of the CNS that are often cited as indicators of a dangerous pro-inflammatory milieu that must be suppressed to achieve successful treatment. Additionally, we have found that GM-CSF treatment reverses astrogliosis, which is also associated with CNS disorders and has been reported to be reversed by GM-CSF treatment that ameliorates spinal cord injury and inhibits glial scar formation ^87, 88^. Furthermore, multiple studies have shown that there is a very high prevalence of cognitive impairment in ARDS survivors, from between 70–100% at hospital discharge, to 46–80% at one year, and persisting in 20% at five years ^89– 91^, which strongly argues for extended treatment with GM-CSF/sargramostim, given its ability to improve cognition in several animal models and reverse cognitive impairment associated with chemotherapy and neurodegeneration.

Because GM-CSF/Sargramostim has been safely and routinely used as a subcutaneous injection treatment and, off-label, as an inhalation treatment, we suggest that this long-FDA-approved drug should be tested for its ability to improve recovery in COVID-19 patients and/or after resolution of infection in severe ARDS survivors to ameliorate potential cognitive deficits. Although compassionate use is possible, it is more important that, either, or a combination, of these two routes of administration in a randomized, pilot trial be used to properly assess the possibility that the innate immune system stimulant, GM-CSF, may reduce morbidity and mortality from COVID-19, as the studies discussed above suggest. Indeed, while an earlier version of this manuscript was being reviewed, a trial of sargramostim in COVID-19 patients with ARDS was announced in Europe using both inhaled/nebulized and/or injected administrations ^92^. We hope that sites in the U.K., the U.S., and elsewhere will be invited to join that study, and welcome comments and data at: https://medschool.cuanschutz.edu/GMCSF-COVID

If GM-CSF/sargramostim proves to be beneficial as a treatment for COVID 19, the fact that the U.S. Department of Health and Human Services maintains a stock of rHuGM-CSF/sargramostim to be used in the event of a nuclear accident may provide short-term resources ^93^.

## Data availability

No data is associated with this article.
