# Supplementary material for: Recruiting the innate immune system with GM-CSF to fight viral diseases, including West Nile Virus encephalitis and COVID-19
Source: F1000Res. 2020 May 11;9:345. [Version 1] doi: 10.12688/f1000research.23729.1 (PMC7359749; doi:10.12688/f1000research.23729.1)
Supplement: Peer review history

# Peer review: Reviewer response for version 1

**DOI:** 10.5256/f1000research.26181.r65612

**Published:** 2020-07-13

**Authors:** Zissis Chroneos

**Affiliations:** 1 Department of Pediatrics, Microbiology and Immunology, Pennsylvania State University College of Medicine, Hershey, PA, USA

The commentary of Potter *et al.* draws parallels from their finding that GM-CSF ameliorates intrinsic and viral neuro-inflammation induced in the course of Alzheimer's disease and West Nile Virus infection, respectively. The authors propose that the application of GM-CSF may have utility for the treatment of COVID-19. Neurotropism for SARS-CoV-2 and other respiratory coronaviruses has been documented, although this is not discussed in the present commentary as a relevant source of targetable neuroinflammation by GM-CSF administration.

 Supporting a beneficial role of GM-CSF, there is an abundance of studies that supplementation of GM-CSF in the airway enhances resistance to viral and bacterial pneumonia and, furthermore, that therapeutic GM-CSF inhalation can stabilize critical illness by macrophages increasing the odds for survival. These beneficial effects are observed locally at a high GM-CSF dosage. It is well-established that the effects of GM-CSF on myeloid cell behavior, activation, and interactions interfacing innate and adaptive immunity are concentration- and source-dependent. Homeostatic functions of GM-CSF at local sites, such as surfactant metabolism by alveolar macrophages may contribute to anti-inflammatory scavenging and repair of tissue damage at a time of need. On the other hand, activated lymphocytes unloading their GM-CSF payload in inflammatory immune synapses during immune remediation against an infectious agent could deliver substantial collateral damage and tissue destruction. To this end, there is an abundance of studies that GM-CSF contributes to adverse inflammation in multiple sterile and non-sterile inflammatory models, autoimmune disease models, and in chronic inflammatory disease caused by chronic environmental exposures such as smoking.

 Buttressing a detrimental contribution of GM-CSF in the development of COVID-19, there are a plethora of clinical trials underway to neutralize GM-CSF signaling. This, however, is evolving against a backdrop of disappointing data that IL-6 blockade in the Sanofi and Regeneron trials were not effective for the treatment of COVID-19. The article could be updated with this latest information, although the effects of GM-CSF in such trials remains to be seen.

 The challenge in designing immuno-modulatory therapies for COVID-19 is the systemic nature of the syndrome. Timing, disease stage, manifestation, and severity, dosage, and route of administration are critical parameters. Significant research effort is, therefore, required to inform the decision making process for GM-CSF supplementation or blockade gave the diverse functions of resident myeloid cell populations in different tissues. A more discerning argument on the compartmental utility of  GM-CSF in preventing cognitive sequelae of COVID-19 is thus warranted.

Is the topic of the opinion article discussed accurately in the context of the current literature?

Yes

Are arguments sufficiently supported by evidence from the published literature?

Yes

Are all factual statements correct and adequately supported by citations?

Yes

Are the conclusions drawn balanced and justified on the basis of the presented arguments?

Partly

Reviewer Expertise:

Mechanisms of infectious and noninfectious inflammatory syndromes of the lung, surfactant protein immune functions, GM-CSF in pulmonary homeostasis

I confirm that I have read this submission and believe that I have an appropriate level of expertise to confirm that it is of an acceptable scientific standard, however I have significant reservations, as outlined above.

# Peer review: Reviewer response for version 1

**DOI:** 10.5256/f1000research.26181.r63287

**Published:** 2020-07-10

**Authors:** W.Sue T Griffin

**Affiliations:** 1 Department of Geriatrics, University of Arkansas for Medical Sciences, Little Rock, AR, USA

In an interesting perspective on immune system-based strategies to combat COVID-19 and its accompanying cytokine storm, Potter *et al.* discuss the relationship of this disease—and particularly, the hyperinflammatory syndrome manifest in the most severe cases—to other disorders. While it may not be surprising to SARS-CoV-2 infections placed vis-à-vis with other viral infections such as West Nile Virus (WNV), some readers may be shocked to see the comparison extended to Alzheimer’s disease (AD). But this is not an unreasonable stretch. The inflammatory hypothesis of AD has been around since the late 1980s, when we and others began to report the elevation of immune-system markers such as interleukin-1 and complement proteins ^1^' ^2^' ^3^' ^4^. We were reluctant to refer to these changes as “inflammation” at the time, as the brain’s partial privilege from immune sequelae ^5^ puts a damper on the *calor, dolor, *and *rubor* classically associated with peripheral inflammation. Nevertheless, the cytokines most closely associated with inflammation now appear to drive every element of AD pathology, as we proposed in the “cytokine cycle” ^6^ (which a more poetic turn of phrase might have rendered “cytokine cyclone” and thus provided the first such “storm”).

 The elaboration of cytokines during neurodegeneration must be presumed to arise from some program that would be otherwise appropriate and adaptive, and several findings indicate that it is modulation rather than wholesale suppression of immune mediators that will be beneficial in AD. Potter *et al.* touch on this in reference to the effects of lipopolysaccharide in mouse models of AD, though they could have helped their case by including the intriguing finding that such stimuli can attenuate amyloid pathology ^7^. Many current therapeutic attempts are aimed at manipulating inflammatory pathways in AD, including the application of GM-CSF (or a clinical preparation known as “sargramostim”) by Potter’s group. The strange career of COVID-19 and the varied effects it has on multiple organ systems suggests a similar need for targeted manipulation of immune responses.

 Evidence clearly indicates that SARS-CoV-2 promotes inflammation by augmenting actions of the innate immune system. In addition to the cytokine storm, the life-threatening form COVID-19 sometimes takes is associated with other inflammatory markers such as C-reactive protein, ferritin, and D-dimers, as well as elevation of the neutrophil:lymphocyte ratio ^8^. It is possible that this reflects attempts by the innate immune system to pick up the slack left by virus’s targeted suppression of acquired immunity via an exquisite ability to suppress interferon-dependent responses ^9^. This provides one more similarity to AD via the changes in immune systems brought about by advanced age, which invariably results in lymphocyte decline and the elevation of inflammatory cytokines as a component of the senescence-associated secretory phenotype.

 Potter *et al.* acknowledge this evidence of an overactive innate response in their criticism of treatments that would suppress the entire immune system, such as steroids and NSAIDS. This latter is also cited in relation to the ADAPT trial of naproxen or celecoxib in AD, halted due to untoward side effects of celecoxcib. However, it is interesting to note, as the authors do, that nondemented subjects who took naproxen for two or three years of the trial had reduced rates of developing AD. Such findings fit well with a VA database retrospective study of ibuprofen and naproxen use among almost 50,000 clinically and neuropathologically confirmed Alzheimer patients and 200,000 non-AD patients. The odds of being in the AD group was reduced from 1.03 to 0.56—i.e., more than 40 percent—among those who took ibuprofen for 5 years or longer ^10^.

 Less compelling are the prospects for effectively guiding the immune system through a SARS-CoV-2 attack with GM-CSF. Potter *et al.* suggest that the success of sargramostim to treating some forms of leukopenia means it will boost lymphocyte actions leukopenia. However, the difference in CD4+ lymphocytes produced by sargramostim treatment was just 22 percent, and this was only after 6 months of treatment ^11^. The vast majority of leukocytes that accumulate after GM-CSF/sargramostim administration are neutrophils, with a considerable number of eosinophils, monocytes, and macrophages; this would seem to be precisely contraindicated in COVID-19. Indeed, Zhou et al. (2020), cited by Potter et al., suggest suppression of GM-CSF among other strategies to restrain the innate immune system.

 Thus, the logical argument for GM-CSF in SARS or COVID-19 is one that should raise red flags; there is a chance that it could worsen outcomes. Improved outcomes with GM-CSF in the WNV model is encouraging. However, it should be noted that WNV is in an entirely different phylum from coronaviruses. Thus, the WNV data may serve best as justification for conducting preclinical studies with a relevant coronavirus model. In the end, it is imperative that no stone is left unturned in the quest for effective treatments for COVID-19 while we suffer without a vaccine. For regardless of how different that acute disease might be from AD and related disorders, there is every reason to believe that the inflammatory ravages triggered by SARS-CoV-2 will set in motion cyclones and cycles that will increase the rates of chronic neurodegeneration.

Is the topic of the opinion article discussed accurately in the context of the current literature?

Yes

Are arguments sufficiently supported by evidence from the published literature?

Partly

Are all factual statements correct and adequately supported by citations?

Partly

Are the conclusions drawn balanced and justified on the basis of the presented arguments?

Partly

Reviewer Expertise:

NA

I confirm that I have read this submission and believe that I have an appropriate level of expertise to confirm that it is of an acceptable scientific standard.

## References

[1] : Complement activation in amyloid plaques in Alzheimer's dementia. *Virchows Arch B Cell Pathol Incl Mol Pathol*.1989;56(4) : 10.1007/BF02890024 259-62 10.1007/BF02890024 2565620

[2] : Brain interleukin 1 and S-100 immunoreactivity are elevated in Down syndrome and Alzheimer disease. *Proc Natl Acad Sci U S A*.1989;86(19) : 10.1073/pnas.86.19.7611 7611-5 10.1073/pnas.86.19.7611 2529544PMC298116

[3] : Complement activation by beta-amyloid in Alzheimer disease. *Proc Natl Acad Sci U S A*.1992;89(21) : 10.1073/pnas.89.21.10016 10016-20 10.1073/pnas.89.21.10016 1438191PMC50268

[4] : Activation of the classical complement pathway in brain tissue of Alzheimer patients. *Neurosci Lett*.1989;107(1-3) : 10.1016/0304-3940(89)90843-4 341-6 10.1016/0304-3940(89)90843-4 2559373

[5] : Functional capacity of solid tissue transplants in the brain: evidence for immunological privilege. *Proc R Soc Lond B Biol Sci*.1985;224(1236) : 10.1098/rspb.1985.0039 375-87 10.1098/rspb.1985.0039 2862633

[6] : Glial-neuronal interactions in Alzheimer's disease: the potential role of a 'cytokine cycle' in disease progression. *Brain Pathol*.1998;8(1) : 10.1111/j.1750-3639.1998.tb00136.x 65-72 10.1111/j.1750-3639.1998.tb00136.x 9458167PMC8098321

[7] : Intrahippocampal LPS injections reduce Abeta load in APP+PS1 transgenic mice. *Neurobiol Aging*.22(6) : 10.1016/s0197-4580(01)00292-5 1007-12 10.1016/s0197-4580(01)00292-5 11755009

[8] : Pathological inflammation in patients with COVID-19: a key role for monocytes and macrophages. *Nat Rev Immunol*.20(6) : 10.1038/s41577-020-0331-4 355-362 10.1038/s41577-020-0331-4 32376901PMC7201395

[9] : Imbalanced Host Response to SARS-CoV-2 Drives Development of COVID-19. *Cell*.2020;181(5) : 10.1016/j.cell.2020.04.026 1036-1045.e9 10.1016/j.cell.2020.04.026 PMC722758632416070

[10] : Protective effects of NSAIDs on the development of Alzheimer disease. *Neurology*.2008;70(19) : 10.1212/01.wnl.0000311269.57716.63 1672-7 10.1212/01.wnl.0000311269.57716.63 18458226PMC2758242

[11] : Phase III study of granulocyte-macrophage colony-stimulating factor in advanced HIV disease: effect on infections, CD4 cell counts and HIV suppression. Leukine/HIV Study Group. *AIDS*.2000;14(4) : 10.1097/00002030-200003100-00012 387-95 10.1097/00002030-200003100-00012 10770541

# Peer review: Reviewer response for version 1

**DOI:** 10.5256/f1000research.26181.r63768

**Published:** 2020-06-23

**Authors:** Sharon Celeste Morley

**Affiliations:** 1 Division of Pediatric Infectious Diseases, Department of Pediatrics, Washington University School of Medicine, St. Louis, MO, USA

This opinion article by Potter *et al.* presents the provocative hypothesis that targeted stimulation of certain immune populations by the administration of GM-CSF could ameliorate certain neuroinflammatory conditions, such as Alzheimer's or West Nile Virus infection, or pulmonary conditions such as COVID.

 There are many references drawn together to support this suggestion. However, there is insufficient clarity in the arguments to introduce the subject to readers unfamiliar with the literature.

 Of largest concern is the casual mixing of very different immunomodulators, without discussion of the various mechanisms of action. The authors discuss steroids, NSAIDS, minocycline, monoclonal antibody therapies, and an experimental therapy called PLX5622 without an adequate introduction to the very different pathways inhibited by these modulators.

 Additionally, the authors do not always distinguish between the different cell types affected. For instance, they comment that GM-CSF might help COVID by improving the "leukopenia" noted in severe cases. However, it is LYMPHOPENIA that marks the most severe cases of COVID, and systemic GM-CSF administration will not help that at all.

 In terms of GM-CSF, the authors do not clearly distinguish between the likely different mechanisms of action when GM-CSF is administered systemically vs via inhalation. It is unlikely, for instance, that inhaled GM-CSF stimulates demargination of PMNs to the same extent that subQ does.

 Finally, the combined discussions of auto-neuro-inflammation (e.g. Alzheimer's), WNV neuroinflammation, and pulmonary inflammation becomes confusing.

 In short, this article would be vastly improved by separating the topic into multiple subheadings, and addressing one sub-topic (e.g. "Immunomodulators in Alzheimer's," "Immunomodulators in WNV,") at a time.

 The information is good. The references are solid. The proposal of using GM-CSF therapeutically to modify inflammation is exciting. But the disorganization of this article currently obscures its strengths, and it should be rewritten for clarity.

Is the topic of the opinion article discussed accurately in the context of the current literature?

Yes

Are arguments sufficiently supported by evidence from the published literature?

Partly

Are all factual statements correct and adequately supported by citations?

Yes

Are the conclusions drawn balanced and justified on the basis of the presented arguments?

Partly

Reviewer Expertise:

Pulmonary innate immunity

I confirm that I have read this submission and believe that I have an appropriate level of expertise to confirm that it is of an acceptable scientific standard, however I have significant reservations, as outlined above.
